# On Proposed National Dental Association

**Published:** 1884-07

**Authors:** B. H. Catching

**Affiliations:** Atlanta, Ga.


Editor Register—*Dear Sir:* Dr. H. W. Morgan, Jr., in your June issue, in a derisive manner, asks questions in regard to the proposed National Dental Association. He sees fit to mark it number two, when there is no intention of forming dual organizations bearing the same name for the same purpose.

To those that he may have persuaded differently this communication is addressed:

The profession in large numbers is calling for an organization based on the plans set forth in a circular addressed to each State Association in the United States. This shows not only the disposition but the time to be most opportune. State after State has elected delegates, and more will do so during this and next month. Success is now assured, and why should any one *kick*, much less a man who is seldom known in meeting outside of his own city. His figures as to representation, like his communication, are flat: 12,000 dentists in the United States, one hundred in the American Association and about the same number in the Southern.

Where are the other eleven thousand and eight hundred? Is there not room, not only for one more, but for two or three associations, according to the above figures.

Now we have too many stay-at-homes. Can we bring out such, not by mouth or pen, but in person? Such is our desire and aim.

We will meet in Washington City, July 22, and there join with the existing National and form an association, not of a section, but of the whole country; not of a few, but of the masses; not to honor a few but to honor the profession.

Sectional associations will go on accomplishing these good purposes. Our duty will ever be to look well to home interests, but while doing this, there is time to be given to the profession at large. A shout to see American dentistry hold and improve the name it has before the world. To stop is to go backward, to go on is to perpetute that which every native dentist must feel proud of. Away with narrow-mindedness and bigotry; down with every thing that is small, up with every thing that is noble. Ambition for self-agrandizement will lower the standard of any calling.

Let us work for a common cause and for a common purpose, and let this be the elevation and perpetuation of American dentistry.

This country, foremost of the civilized world, will not be lacking when the time comes to show that it will honor the profession that has honored the country. B. H. CATCHING,

Atlanta, Ga., June 14, 1884.

